# Design and Evaluation of Magnetic Hall Effect Tactile Sensors for Use in Sensorized Splints

**DOI:** 10.3390/s20041123

**Published:** 2020-02-19

**Authors:** Dominic Jones, Lefan Wang, Ali Ghanbari, Vasiliki Vardakastani, Angela E. Kedgley, Matthew D. Gardiner, Tonia L. Vincent, Peter R. Culmer, Ali Alazmani

**Affiliations:** 1 School of Mechanical Engineering, University of Leeds, Leeds LS2 9JT, UK; 2 Department of Bioengineering, Imperial College London, London SW7 2AZ, UK; 3 Kennedy Institute of Rheumatology, University of Oxford, Oxford OX3 7FY, UK

**Keywords:** tactile sensors, soft sensing, force sensors, Hall effect sensor, magnetic field, hyperelastic elastomer, silicone rubber, calibration, hand splint

## Abstract

Splinting techniques are widely used in medicine to inhibit the movement of arthritic joints. Studies into the effectiveness of splinting as a method of pain reduction have generally yielded positive results, however, no significant difference has been found in clinical outcomes between splinting types. Tactile sensing has shown great promise for the integration into splinting devices and may offer further information into applied forces to find the most effective methods of splinting. Hall effect-based tactile sensors are of particular interest in this application owing to their low-cost, small size, and high robustness. One complexity of the sensors is the relationship between the elastomer geometry and the measurement range. This paper investigates the design parameters of Hall effect tactile sensors for use in hand splinting. Finite element simulations are used to locate the areas in which sensitivity is high in order to optimise the deflection range of the sensor. Further simulations then investigate the mechanical response and force ranges of the elastomer layer under loading which are validated with experimental data. A 4 mm radius, 3 mm-thick sensor is identified as meeting defined sensing requirements for range and sensitivity. A prototype sensor is produced which exhibits a pressure range of 45 kPa normal and 6 kPa shear. A proof of principle prototype demonstrates how this can be integrated to form an instrumented splint with multi-axis sensing capability and has the potential to inform clinical practice for improved splinting.

## 1. Introduction

Orthotics are widely used in medical practice to modify the load-bearing characteristics of the body. In particular, splints focus on the fixation and offloading of pathological joints to prevent further damage and assist in recovery [1]. Thanks to their non-invasive nature, splints are used as a conservative method for management or treatment in diseases such as arthritis, with more severe cases eventually requiring surgical intervention [2]. While splints are widely used, the evidence behind their efficacy is limited [3] indicating a need for biomechanical analysis of their effects. To inform this there is the potential to integrate tactile sensing within splinting devices to both measure and characterise the abnormal forces and pressures found in different diseases. Such devices could be used to assist both diagnosis and the monitoring of disease progression [4].

Previous studies into instrumented splints have investigated the pressure distribution within different orthotic devices, in order to analyse the interactions between the splint and the body. Most devices utilise single-axis sensors to monitor the contact pressure between the device and skin. Earlier research in this area made use of force sensing resistors fixed on the inner surface of plaster casts. Laufer et al. utilised an array of seven sensors to measure the intra-cast pressure during the application and removal of a plaster cast [5]. More recently, a similar technique was used by Tuan et al. to monitor the varying pressure between soft tissues and bone protrusions in the arm [6]. Both were able to detect abnormal cast pressures during the fitting procedure and had the potential to be used for the detection and prevention of some pressure sores. Pressure levels within the arm casts were at maximum 1 kPa under normal conditions, rising to a maximum 20 kPa in abnormal circumstances.

Other groups have used pressure sensing within splints to measure disease progression and quantify the success of different treatment methods of musculoskeletal diseases. Giesberts et al. used a tactile sensing unit, applied to the inner face of a cast and splint, to measure the correction of club-foot and Dupuytren disease [7,8]. Both devices monitored the pressure of the orthotic on the underlying skin over a period of up to 12 hours, indicating a 95% reduction in applied pressure over the study. A benefit of the custom load sensor used in the studies was the ability to control the overall geometry of the sensor. The commercial force sensing resistors used in earlier studies are typically very low profile, but being commercial products are limited to specific sizes and geometries, and are generally better suited to analysing hard contact [9,10]. Giesberts’ custom sensor was designed to a specific geometry to fit between the orthosis and skin; being thick enough to ensure constant contact throughout the study. Again the single axis nature of the sensor limits the device to specific use cases where only normal force is required. One problem with these devices is the limitations caused by the use of single-axis force sensing. Studies have shown that the monitoring of shear stresses are equally as important in formulation of pressure-based skin diseases [11,12] and as such must be measured to gain a full understanding of the biomechanical interaction between the splint and body.

One modality which offers a promising solution to splint instrumentation is Hall effect-based tactile sensing. Through use of a moving magnet and a Hall effect sensor as a transducer method, a compliant and high performance tactile sensor may be produced. This method also allows for the electronics to be located on the exterior of the splint without direct contact to the sensing face. Wang et al. [13] presented a design methodology to produce a magnetic based tactile sensor, establishing a manufacturing protocol and calibration method to develop subsequent prototypes. Further to this, de Boer et al. [14] focused on the optimisation of this prototype, using finite element (FE) models to explore the magnetic and material properties of the MagOne. Computational optimisation of the design indicated the complex non-linear responses between applied forces and subsequent changes in magnetic field. This also highlighted that small variances in the geometric parameters of the elastomer could yield a large change in sensitivity in both normal and shear forces.

In this paper we aim to produce a three-axis tactile sensor for use within a thumb immobilisation splint. This work furthers our previous research on the design of Hall effect tactile sensors [13,14], allowing for design parameters to be selected on the underlying interaction between the Hall Effect sensor and magnet, rather than focusing on the elastomer design. To identify appropriate design parameters for the sensing system, we identify a series of system requirements and then use validated FE models to explore the mechanical and magnetic design space. We present this approach in Section 3 and use it to identify a sensor design for integration within a hand splint. A prototype of this design is produced and then experimentally evaluated in Section 4.

## 2. System Description

**Figure 1:**
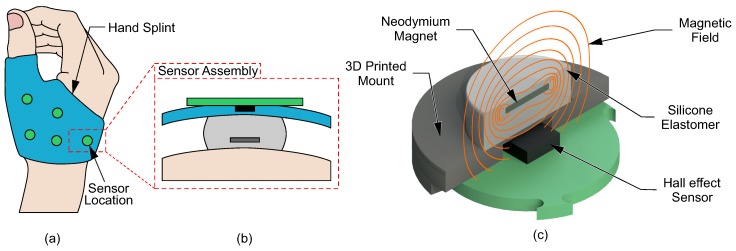
(**a**)The sensorised splint concept, (**b**) Placement of the sensor in the proposed splinting method, and (**c**) Components of the soft Hall effect tactile sensor: A neodymium magnet, silicone elastomer, and a three-axis Hall effect sensor.

This research concerns an instrumented hand splint which integrates a series of Hall effect-based tactile sensors, as shown in Figure 1a,b. In this concept, initially presented by Wang [13], the electronics of the sensing elements can be integrated and encapsulated with the splint body, with the elastomeric sensing element protruding and contacting with the surface of the hand. The sensing elements can be located at specific locations of clinical, or biomechanical, interest to monitor multi-axial contact forces between the hand and splint. The sensor consists of three main parts: A three-axis Hall effect sensing chip (MLX90393, Melexis); an elastomer body; and an embedded magnet (Figure 1b). Under the application of force to the upper surface of the sensor the elastomer layer deforms, causing the magnet to move. This change in position ($U_{x,y,z}$) of the magnet is detected in three dimensional (3D) space through the changing magnetic field vector sensed by the chip ($B_{x,y,z}$). The elastomer body modulates this movement, and allows the calibration of position change directly to force ($F_{x,y,z}$). The use of an elastomer to modulate the magnet’s displacement causes the output force range and sensitivity to vary according to the elastomer’s mechanical properties. In this way, a soft elastomer will allow more movement, limiting the force range but increasing maximum sensitivity; whereas a hard elastomer will limit movement, increasing range and reducing sensitivity.

## 3. System Design

### 3.1. Sensor Requirements

The integration of tactile sensing to the splint–skin interface imposes a series of requirements which must be met to allow for accurate pressure sensing and to ensure the functionality of the splint is not compromised. The main factor is sensor thickness; if too thin, the sensor risks insufficient or incomplete contact with the skin surface, causing error in the measurement; too thick, the sensor will protrude into the skin and prevent the rest of the splint surface from stabilising the joint. For this, a thickness constraint was applied to prevent the inclusion of sensors from modifying the functionality of the splint. We used the thickness constraint applied by Giesberts et al. with a maximum sensor thickness of 3 mm [8]. This thickness, while larger than some other tactile sensors, allows for a higher range of deformation and assists in keeping the sensor in constant contact with the hand, reducing the effects of bad fitting observed in some splint designs [15]. The second requirement is the pressure range of the sensor. There is currently little information reported in the literature on the typical pressures which might be observed at the splint–skin interface, and to the best of the authors knowledge, the shear stresses at this interface have not been reported. From current literature, the standard pressures experienced are around 1 kPa for upper limb splints, rising to around 20 kPa under abnormal loading [5,6,8]. This range may vary based on the placement of sensors, with differing forces experienced around anatomical landmarks, however future research will be need to confirm this. To cover this full range with a safety factor of 50%, a minimum sensor range of 30 kPa in pressure was established.

### 3.2. Identifying Sensor Design Parameters

Based on the sensing principles explored in Section 2, it may be seen that there are two main configurable components that affect the sensitivity of the sensor: the magnetic field surrounding the embedded magnet, as well as the structure and geometry of the elastomer body. This offers several design parameters which may be modified to vary the behaviour of the sensor. The first set of design parameters focus around the magnet used in the sensor. The magnet’s geometry, size, and type all affect the field strength and shape, thereby affecting the measured response. For this application, disc magnets were selected as the circular shape caused an axially-symmetric field around the magnet allowing for an identical response in all shear directions. N42 grade neodymium was selected as the magnetic material providing a strong field in a compact size. The disc radius and thicknesses were selected as a range of commercially available sizes, with magnet radius of 2, 4, and 6 mm, thicknesses of 0.5, 1, and 2 mm (Table 1). These discrete geometries form a design constraint for the system which is investigated in Section 3.3.

The elastomer response is affected by two main parameters: geometry, and material. The elastomer selected for the sensor was Ecoflex 00-30 (Smooth-On Inc., Macungie, PA, USA) with previous research and preliminary experiments indicating that the material properties were well suited for use in hand splinting [16]. The total sensor thickness was limited to 3 mm, however the embedded magnet geometry would vary the thickness of elastomer available for compression. A 0.5 mm layer of elastomer was left above the magnet which was required to encapsulate the magnet with sufficient robustness, yet was thin enough to reduce the effects on the sensitivity of the sensor [13]. From these elastomer dimensions an available ’working area’ was established for each magnet thickness, signifying the amount of movement that may occur. An upper limit of compressive strain was set at 50% as a typical working compressive strain for silicone elastomers [17], lower than the 75% typical maximum strain used in compressive material tests [18]. This set a maximum travel limit for each thickness of magnet: 1 mm for the 0.5 mm thickness, 0.75 mm for the 1 mm thickness, and 0.25 mm for the 2 mm thickness. For horizontal displacement, it was assumed that in typical function the horizontal movement of the magnet would never be greater than the vertical displacement. This leads to a triangular ’working area’, signifying the typical limits of magnet’s movement. To compare the different sensor geometries, ratios of both the geometry (Thickness : Radius) and resultant pressures (Normal : Shear) were calculated.

By imposing the design constraints and considerations discussed above, the design process involves identifying appropriate parameters for the sensor’s magnet size and elastomer dome geometry. Our approach uses FE simulations to explore the magnetic and mechanical design space. The magnetic simulation is used to identify an effective workspace for the sensor based on a particular magnet size by defining the physical area which can be sensed with sufficient sensitivity within the operating limits of the three-axis Hall effect sensor. To achieve this, the mechanical simulation was used to develop the elastomer dome design by exploring the relationship between the Thickness:Radius ratio and the resultant sensing range, ensuring in particular it satisfies the requirement to accommodate normal pressures of 20 kPa.

### 3.3. Investigation of Magnetic Design Parameters

**Figure 2:**
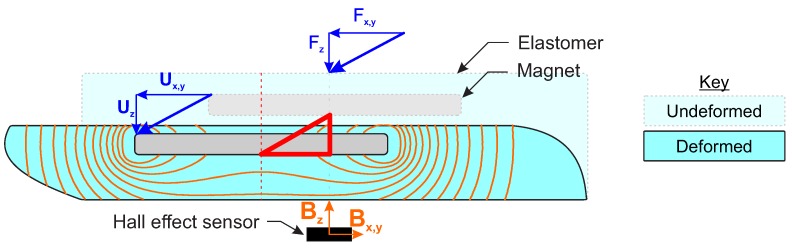
Schematic of the soft Hall effect tactile sensor, indicating the associated measurement parameters: $F_{x,y,z}$—Applied force; $U_{x,y,z}$—Sensor Surface Displacement; $B_{x,y,z}$—Measured magnetic field at the Hall effect sensor (black rectangle); The triangular workspace of the sensor is indicated in red.

An FE model of the magnet was developed within a Multi-Physics FE environment (COMSOL Multiphysics 5.2, Magnetic Fields No Currents module, COMSOL Inc, Burlington, MA, USA) to calculate the magnetic field vectors at all points around the magnet. It was not essential for the magnetic field calculation to be included in the overall model of the sensor, as displacement of the magnet has no effect on the field shape or strength in the reference frame of the magnet (Figure 2). Similarly, owing to the rotational symmetry of the cylindrical magnet selected, the simulation could be simplified to a two dimensional axisymmetric model environment to increase the computational efficiency. For this, the two components of magnetic flux on the horizontal plane, $B_{x,y}$, were combined to form a radial vector, $B_{r}$. (Equation (1)).

(1) $$B_{r}=\sqrt{B_{x}^{2}+B_{y}^{2}}$$

**Figure 3:**
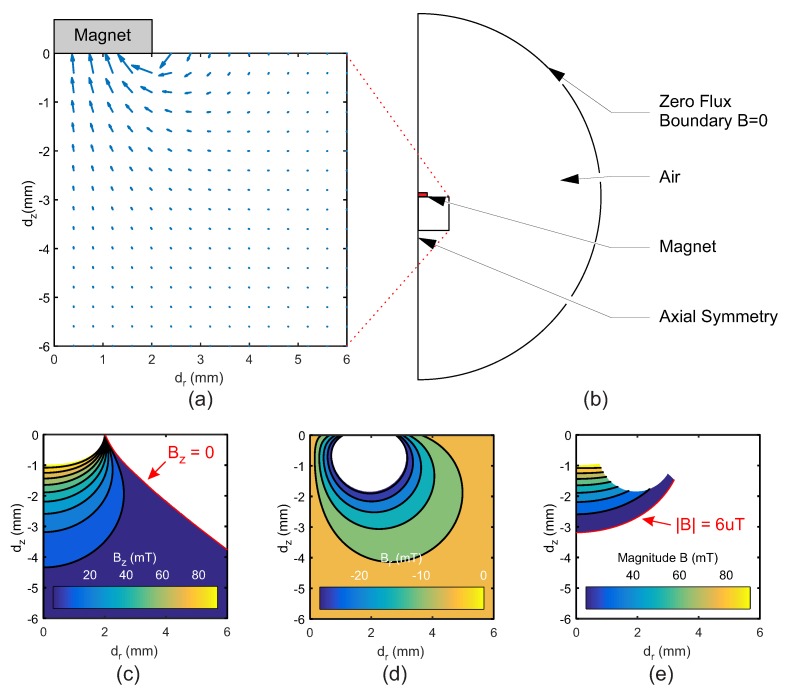
(**a**) Magnetic field vectors in an area of 6×6 mm below the simulated magnet, used in the measurable area definition; (**b**) Geometry of the magnetic field simulation, indicating the domains and boundary conditions used; Areas of sensitivity in which the Hall effect sensor is in range in (**c**) $B_{z}$ (also indicating the inversion line where $B_{z}$ = 0); (**d**) $B_{r}$; and (**e**) Combined limits, magnitude (indicating the minimum accepted field magnitude at 6 uT). $d_{z}$ and $d_{r}$ represent the vertical and horizontal movement of the magnet, respectively. $d_{r}$ = 0 is the axis of rotational symmetry of the disc magnet.

The magnet was treated as a rectangle of length and height equal to the radius and thickness of each tested magnet. The domain had a permanent magnetisation condition applied, with remnant flux density of 1.3 T [19]. This was enclosed within a semi-circular domain of air (radius 50 mm) to the field to propagate, with a zero flux boundary condition applied to the outer circumference (Figure 3b). Magnetic field data ($B_{z},B_{r}$) was output at each node a 10 μm grid in and area of 6 × 6 mm below the magnets bottom face (Figure 3a).

The output data was processed with MATLAB (Mathworks Inc., R2016a, Natick, MA, USA) to assess the various displacement ranges possible in the prototypes. This produced regions within which the Hall effect sensor may be placed while still within sensing range of the magnet’s field. Owing to the fixed position of the magnet there should never be a large rotation to cause the polarity of the field’s vertical component to flip, ensuring no change in the field polarity. The sensor output data was modified to measure a single polarity of the magnetic, such that $B_{z}>0$. This applies an additional constraint to the sensor, in that it must be located between the two inversion lines of the field (where $B_{z}$ = 0). To find the optimal displacements between the sensor and magnet, areas outside of the sensor range were removed. This included both areas where field strength ($B_{z,r}$) were greater than the sensor saturation point and areas where $B_{z}<0$ (Figure 3c,d). Next, a lower bound for the sensor needed to be established. For this, the Earth’s latent magnetic field was utilized. A lower boundary was set where the field was equal to 100 times the maximum strength of the Earth’s magnetic field (Figure 3e). This ensured that the maximum effect of the external field on the sensor was no more than 1% of the sensed magnitude, thereby reducing error in the final measurement when the sensor was moved to a different orientation.

**Figure 4:**
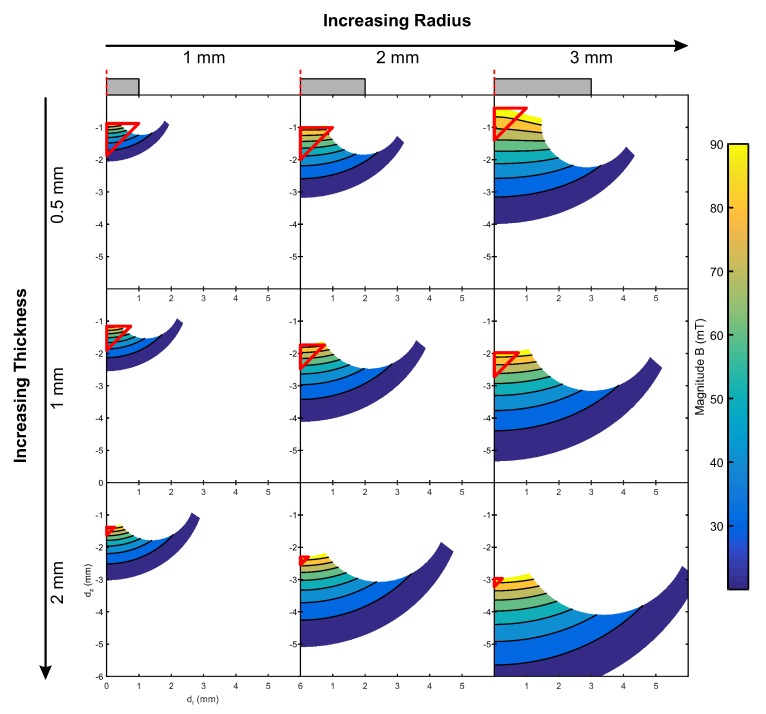
The changing size and position of the “measurable area” in the magnetic field with both magnet thickness and radius. Working areas for the magnets are indicated as red triangles. The working area is located at the point closest to the magnet, where the entire working area is contained within the measurable area, ensuring maximum sensitivity. $d_{z}$ and $d_{r}$ represent the vertical and horizontal movement of the magnet, respectively.

Figure 4 presents the calculated magnetic field strength from the two dimensional axisymmetric FE simulation, with the upper and lower bounds in $B_{z}$ and $B_{r}$ implemented. The field strength is presented as contour lines (T). The nine permutations of magnet size from Table 1 are presented and indicate the changing shape of the “measurable area” with magnet size. The size and position of the measurable area of magnetic field for a range of magnetic configurations. The measurable area varies as a function of magnet thickness and radius, as defined in Section 3.2. A working area is defined for each configuration, shown as a red triangle positioned at the point closest to the magnet where the entire working area is contained within the measurable area to ensure maximum sensitivity. From this analysis, the 2 mm radius, 0.5 mm thickness magnet was selected for mechanical analysis, as it showed the highest usage of the measurable area under working conditions.

### 3.4. Investigation of Physical Design Parameters

**Figure 5:**
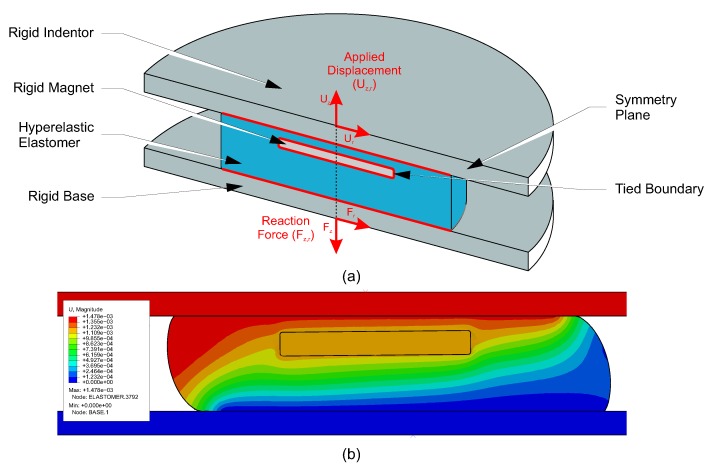
(**a**) Schematic model used in the elastomer mechanics simulation, indicating the main components, and boundary conditions applied. The symmetry boundary conditions were applied across the full z-r plane, red boundaries signify a tied boundary condition. Displacement was applied and reaction force was measured from reference points along the central axis of the model; and (**b**) Typical output from the finite element simulation, contours represent displacement (*U*) magnitude.

An FE model was produced to analyse the structural mechanics of the sensor. The simulation was used to evaluate the mechanical response of these elastomer configurations, thus enabling an appropriate geometry to be selected for the application. A full 3D representation of the sensor and indentation plates was produced for the simulation. In these models a vertical ($d_{z}$) and radial ($d_{r}$) displacement are applied to the top surface of the sensor (Figure 5a), and so the model was not axially symmetric. However, symmetry is present in the vertical plane in the direction of the applied shear, and so a symmetry condition was applied to reduce the overall complexity of the model. For the material properties of the elastomer, a third order incompressible Ogden model was selected based on existing research into the compressive properties of Ecoflex 00-30 [18]. As the stress across both the magnet and indentors were inconsequential for the final sensitivity of the sensor, they were modelled as rigid domains. The elastomer was tied to the surfaces of both magnet and indentors (Figure 5a) such that no slip or detachment occurred during simulation. A contact condition was also established between the elastomer and indentors to contain the expanding outer surface of the elastomer under higher stresses. The initial thickness of the elastomer layer below the magnet was selected from the output of the magnetic simulations to ensure the travel of the magnet covered at least 50% of the available signal from the sensor, leading to the selection of a 2 mm thickness base.

To apply the forces to the sensor, a prescribed displacement boundary condition was added to the upper indentor. This allowed the sensor to be loaded in the same manner as the calibration system used in Section 4.1. The maximum displacement in both $d_{z}$ and $d_{r}$ was selected to be equal to the maximum range of vertical movement, taken from the working areas defined in Section 3.2. The reaction force ($F_{zr}$) was recorded to define the force range of the sensors. Five sizes of elastomer were simulated each with total thickness 3 mm inclusive of the embedded magnet, with radius ranging from 4–12 mm (Table 2). These values were selected as the reaction forces of the elastomer were unknown, so the wide range of values would allow a relationship between input geometry and output force to be established. The simulation was validated using the loading regime described in Section 4.2. The validation indicated consistency between the simulated and experimental results, the mean normal and shear pressure ranges in the experiment were 44.82 ± 0.08 and 6.11 ± 0.03 kPa. This equated to a difference in pressure of +2.88% for the normal range and +5.31% for the shear range.

An example of the output of the FE mechanical simulations is shown in Figure 5b. In this, the magnitude of displacement is shown across the full cross section of the sensor at top indentor displacement $U_{z}$ = −1 mm and $U_{r}$ = 1 mm. The negative vertical displacement causes compression of the elastomer in the negative z direction, while expanding equally in both directions in the x direction owing to the Poisson effect. The horizontal displacement causes shear on the top surface of the sensor, causing a tilting of the displacement in the direction of shear.

**Figure 6:**
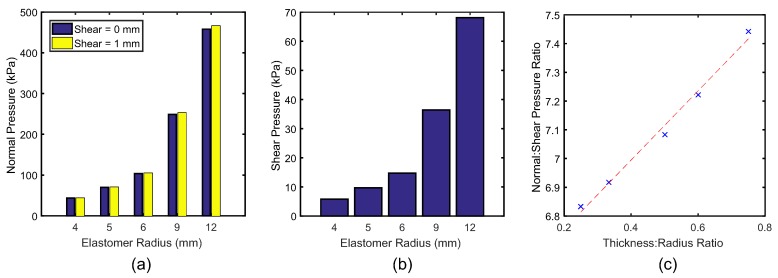
Maximum force output at 1 mm strain in (**a**) normal pressure and (**b**) shear pressure. (**c**) The effects of geometry ratio change on the output force ratio, a linear relationship is indicated

Figure 6 presents the pressure outputs from the mechanical simulation for different sizes of elastomer. In Figure 6a,b, both normal and shear pressure are shown to increase with sensor radius, from 43.6 and 5.8 kPa at 4 mm radius to 458 and 68 kPa at 12 mm radius, respectively. Figure 6c indicates a linear relationship between the geometry and force outputs when examined as a ratio (Thickness:Radius and Normal:Shear Pressure)($R^{2}$ = 0.98). The 4 mm radius sensor offered a range closest to that of the initial prescribed requirement, and was therefore selected for the manufactured prototype.

## 4. System Evaluation

A prototype of the sensor intended to be used in the splint was produced to the selected geometry and magnet size. The sensor was manufactured using Ecoflex 00-30 and N42 Neodymium magnet. The silicone was cast in a 3D printed mould and left to cure at room temperature. The magnet was placed into the cavity in the top surface of the sensor and were fixed with cyanoacrylate glue. A second layer of elastomer was applied to fill the remaining cavity, embedding the magnet 0.5 mm below the top surface. The sensors were then mounted on 3D printed mounts above a custom PCB to prevent movement under loading. The custom PCB was designed to be a 12 mm diameter circle, with the 3×3×1 mm Hall effect sensor in the centre of one face. This was produced for ease of connection in the prototype, however may be further miniaturised for future versions.

**Figure 7:**
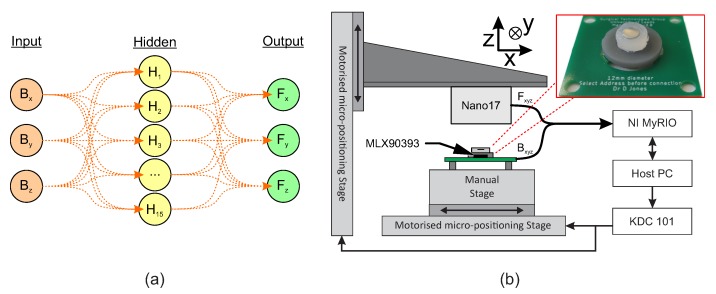
(**a**) Structure of the two-layer neural network used in the calibration; and (**b**) The multi-axis loading test setup , indicating two of the three linear stages used in the calibration. A third stage acts into the plane of the diagram to provide y-axis movement. A real-time measurement device is used to record magnetic field, applied force, and encoded position of the indentor. Inset: The prototype sensor used in calibration with 4 mm magnet embedded.

The sensors were mechanically loaded using a custom three-axis loading platform (Figure 7b) consisting of three linear servomotor stages (MTS50/M-Z8, KDC101, ThorLabs, Newton, NJ, USA) and a six-axis F/T sensor (Nano17, ATI IA, Apex, NC, USA). The system allowed compression and shear rates of up to 2.4 mm/s, with a travel range of 50 mm, a repeatability of 1.6 μm and a minimum repeatable incremental movement of 0.8 μm. The F/T sensor had a force measurement range of ±35 N in the z-axis and ±25 N in both the x and y axes, with minimum resolution 6 mN. The system applies load to the sensor surface though prescribed displacement among each of the 3 axes independently. Motion in two stages may be synchronised to allow non-central loading of the sensor (i.e., x- and z-axis simultaneously). The resultant forces on the sensor are measured by the F/T sensor and recorded through a custom data acquisition system (MyRIO, LabVIEW, National Instruments, Austin, TX, USA). The prototype was subjected to the loading regime as the simulation, compressing the sensor vertically by 1 mm, then shearing by 1 mm.

### 4.1. Sensor Calibration

Neural network fitting was used to characterise the non-linear relationship between the measured three-axis magnetic field and the associated three-axis load. This forms the basis for the sensor calibration as shown in Figure 7. The networks were trained using the Matlab *NNfit* toolbox using a Levenberg-Marquardt backpropagation algorithm for training [20]. The resultant two layer feed-forward network (Figure 7a) has 15 neurons in the hidden layer and a hyperbolic tangent activation function. Training data was recorded using a three-axis loading regime using the calibration system shown in Figure 7b. The regime applied a prescribed displacement to the upper surface of the sensor, recording force data in a 0.8 × 1.6 × 1.6 mm grid, through the values of $U_{z}$ from 0 to 0.8 mm and $U_{xy}$ from −0.8 to 0.8 mm at increments of 0.05 mm. The calibration was then tested under the same loading regime, comparing the input load to the calibrated output.

**Figure 8:**
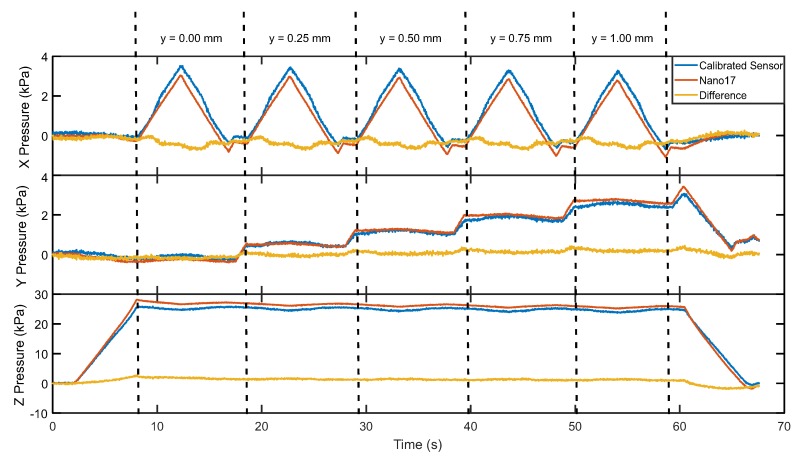
Response of the splint sensor prototype vs. Nano17, with load variation in three axes.

Figure 8 presents a comparison of the reference load cell output and the value from the calibrated sensor. The sensor was compressed to a maximum of 30 kPa, and sheared to a maximum of 3.4 kPa in the x and y dimensions, meeting the initial range requirement. The Root Mean Square (RMS) error in each axis was 0.36 (11%), 0.15 (4.5%), and 1.35 (4.8%), for the x, y and z measurements respectively. The RMS error in the x direction is greater than that of z and y, possibly because of inconsistencies in the calibration method. This is likely due to time related effects such as creep and hysteresis in the elastomer, as the x axis is constantly in motion throughout the test. RMS error in the z direction is consistent throughout the whole test. RMS in the y direction increases slightly with increasing displacement in y, but is consistent throughout each x sweep.

### 4.2. Splint Sensor Prototype

**Figure 9:**
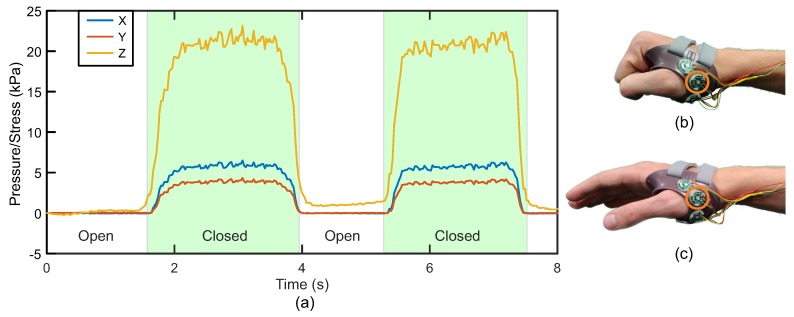
(**a**) Response of the prototype from a single sensor on the sensorised splint over two hand closing actions (closed hand indicated in green), (**b**) closed hand, and (**c**) open hand. The orange circle indicates the sensor from which the measurements were made.

To indicate the sensor’s effectiveness within a splint, four sensors were manufactured and calibrated. These sensors were integrated into a commercially available hand splint (Thumb Brace, Push Ortho, The Netherlands). A single healthy participant wore the splint, opening and closing their hand (Figure 9b,c) multiple times in order to generate forces at the splint–skin interface. Pressures were measured and recorded at 50 Hz using a custom data acquisition system. Pressures of around 20 kPa and shear stresses of around 5 kPa were observed while the hand was closed, similar to the pressures reported from literature. While the individual sensor sizes are relatively large in this prototype, there is significant opportunity to miniaturise the system, through optimisation of sensing electronics and the elastomeric layer. This work will be informed by preliminary testing to better understand typical shear loading regimes which are currently not well understood.

## 5. Discussion

The investigation into the magnet sensitivity indicated two main design features that will assist in magnet selection for individual purposes. First, the sensor resolution and sensitivity is heavily dependent on the magnet radius. As seen in Figure 4, the magnet radius has a much greater effect on the shape and size of the measurable area than the magnet thickness. When coupled together, the radius may first be used to set the limits on the range of motion required by the final sensor, and then to vary the thickness to define the position of the working area of the sensor.

Secondly, the saturation point of the sensor, when combined with variation in the magnet thickness, can be utilised to deduce the optimal position of the magnet above the sensor. If the magnet is placed such that the minimum distance between the magnet and Hall effect sensor (under the maximum normal force) is equal to the point at which the sensor saturates, the full range of the sensor will be at the highest possible resolution at all points. This means that each magnet and sensor setting combination will yield an individual “measurable area”. Outside of this area further magnet movement will either: saturate the sensor in one or both dimensions, causing failure of the calibration; or sensitivity will decrease causing progressively larger error as the applied magnetic field tends towards zero.

The geometry of the elastomer is important in defining the “working area” of the sensor, where movement of the magnet will occur under standard usage. For the sensor to perform optimally, the functional area of the sensor must be maximised in relation to the measurable area below the magnet face. The displacement limit of 1 mm was applied to limit the applied strain to 50%, after which the hyperelastic model would break down owing to the strain surpassing that of the experiment used in the model generation.

The radius/thickness size ratio of the sensor affects the normal/shear pressure ratio. This offers the potential for further optimisation when more is known about the shear interactions of the splint. If shear is shown to be a more important factor in the usage of splinting devices, the sensor geometry may be changed to offer either a greater range or resolution in the shear direction, thereby improving sensor performance. Further to this, if elastomers with similar mechanical responses to Ecoflex 00-30 are used to produce the sensor, the relationship between geometry and pressure range should remain consistent allowing an initial estimation of a sensor’s properties to reduce the number of design iterations required in future research. The experimental validation of the sensor presented in Section 3.4 indicates the validity of these simulations with output values falling within a 6% bound of the validated forces. As such, the usage of simulations has accelerated the sensor design process, enabling a detailed exploration of the design space in order to select appropriate parameters to meet the system requirements.

The sensor prototype can be seen to respond with comparable accuracy to the commercial load cell used as a reference measurement showing that the sensor is capable of load measurement within the 30 kPa range set out as a requirement in Section 1. The magnitude of the sensor output is consistently lower than the the reference in the y and z axes, but higher in the x, which is likely due to viscoelastic effects during the sensor calibration, where the position was rapidly swept through the x-axis while sweeping slower through the y and z axes, allowing for stress relaxation in the latter, causing the offset in force. While this is the case, the overall design method for the sensor remains sound, but further improvement on the calibration methodology is required to reduce the effects of viscoelaticity on the final calibration. The sensor allows force measurement in a form factor that current commercial load cells of similar sensitivity cannot achieve, showing the sensor’s great potential for the measurement of multi-axis pressures in the splint–skin interface, allowing for consistent contact and measurement. Although this study focuses on splinting, the sensor may also be designed and applied in other medical applications, such as grip sensing or other orthotic interface measurements.

## 6. Conclusions

This study presented a design method for the development of tactile sensors for upper limb splinting. Using FE simulations, both the sensitive areas of the sensor and the predicted output forces were obtained for several geometries. A prototype was produced from the optimal design, which was then calibrated and experimentally characterized, indicating that it met the requirements for in-splint sensing. The sensor may serve as a valuable tool for research into patient splint interaction, allowing for the analysis of multiple dimensions of contact pressure.

## Figures and Tables

**Table 1:** Parameters of Magnet geometry in the magnetic field simulation, all combinations of parameter were used.

Magnet Radius (mm)	Magnet Thicknesses (mm)
1	0.5
2	1
3	2

**Table 2:** Parameters of elastomer geometry used in the mechanical simulation.

Elastomer Radius (mm)	Total Thickness (mm)	Thickness:Radius Ratio
4	3	0.75
5	3	0.6
6	3	0.5
9	3	0.33
12	3	0.25
